# Mixing Time Effects on the Dispersion Performance of Adhesive Mixtures for Inhalation

**DOI:** 10.1371/journal.pone.0069263

**Published:** 2013-07-02

**Authors:** Floris Grasmeijer, Paul Hagedoorn, Henderik W. Frijlink, H. Anne de Boer

**Affiliations:** Department of Pharmaceutical Technology and Biopharmacy, University of Groningen, Groningen, The Netherlands; Massey University, New Zealand

## Abstract

This paper deals with the effects of mixing time on the homogeneity and dispersion performance of adhesive mixtures for inhalation. Interactions between these effects and the carrier size fraction, the type of drug and the inhalation flow rate were studied. Furthermore, it was examined whether or not changes in the dispersion performance as a result of prolonged mixing can be explained with a balance of three processes that occur during mixing, knowing drug redistribution over the lactose carrier; (de-) agglomeration of the drug (and fine lactose) particles; and compression of the drug particles onto the carrier surface. For this purpose, mixtures containing salmeterol xinafoate or fluticasone propionate were mixed for different periods of time with a fine or coarse crystalline lactose carrier in a Turbula mixer. Drug detachment experiments were performed using a classifier based inhaler at different flow rates. Scanning electron microscopy and laser diffraction techniques were used to measure drug distribution and agglomeration, whereas changes in the apparent solubility were measured as a means to monitor the degree of mechanical stress imparted on the drug particles. No clear trend between mixing time and content uniformity was observed. Quantitative and qualitative interactions between the effect of mixing time on drug detachment and the type of drug, the carrier size fraction and the flow rate were measured, which could be explained with the three processes mentioned. Generally, prolonged mixing caused drug detachment to decrease, with the strongest decline occurring in the first 120 minutes of mixing. For the most cohesive drug (salmeterol) and the coarse carrier, agglomerate formation seemed to dominate the overall effect of mixing time at a low inhalation flow rate, causing drug detachment to increase with prolonged mixing. The optimal mixing time will thus depend on the formulation purpose and the choice for other, interacting variables.

## Introduction

Lacey was one of the first to describe the mixing process as the creation of disorder by allowing chance to determine the positions of the particles. With increased mixing time these chance effects accumulate, eventually resulting in a more or less stable equilibrium of maximum disorder [1]. The theoretical ‘random’ mixing process addressed by Lacey excludes particle interaction phenomena and is therefore fundamentally different from the ‘ordered’, or rather, ‘total’ mixing process that best describes the formation of adhesive mixtures for inhalation [2–4]. In both instances the role of mixing time is essentially the same, however, in that it allows chance effects to accumulate and therefore determines the extent to which certain processes within the mixture take place.

On blending of adhesive mixtures for inhalation several such processes can be distinguished. For example, it has been shown that drug agglomerates which are present in the starting material will be broken up [5,6] and evidence suggests that at the same time new, less coherent agglomerates can be formed [7]. Furthermore, the inhomogeneous nature of lactose carrier surfaces allows for redistribution of drug particles to occur between surface sites with a different binding activity or sites with a different capacity to offer sheltering to drug particles from the redistribution forces during further mixing [2,4,8,9]. In addition, repeated press-on forces and possibly triboelectrification effects may gradually increase interaction forces between drug and carrier particles [10–12]. Lactose fines might also be generated by attrition of the carrier, but this process seems to be restricted to high shear blending operations [13,14].

The three processes that are expected to principally affect the dispersion performance of adhesive mixtures and that are therefore subject of this study can be summarised as agglomeration, distribution and press-on processes, respectively. They each may alter the dispersion performance of the mixture in a different direction and with a different magnitude. Therefore, it is to be expected that the overall effect of mixing time on the dispersion behaviour of any carrier-based inhalation formulation is primarily related to the obtained balance between these three principal processes. This also implies that any variable that alters this balance may interact with the effect of mixing time.

The carrier size fraction, the drug concentration and the flow rate were previously identified as variables that can interact with the effect of mixing time on drug detachment during inhalation [15,16]. These variables either directly affect the different processes during mixing by influencing the potential and propensity for drug redistribution and (de-) agglomeration or the magnitude and efficacy of press-on forces, or they affect the significance of their occurrence in relation to drug detachment by altering the dispersion efficacy. For example, the agglomeration behaviour of drugs in adhesive mixtures was shown to be proportional to the carrier size fraction, or more specifically, the interparticulate pore size of the carrier powder bed [7,17]. Furthermore, the significance of a change in drug particle mass (agglomerate size) in relation to drug detachment from lactose carriers during inhalation was found to decrease with increasing dispersion efficacy (e.g. with higher flow rates through the inhaler) [18]. Therefore, for a given drug-carrier combination agglomerate formation is most likely to be a dominant process determining the effect of mixing time on drug detachment when coarse carrier particles are used and dispersion tests are performed at a low flow rate. Another variable that may change the balance of the different processes during mixing and could therefore interact with the effect of mixing time is the type of drug: the balance of intrinsic cohesive to adhesive interaction energy in combination with lactose can be different between drugs, and therefore, so may be their propensity towards agglomeration [19].

Mixing is unquestionably the most important unit operation in the formulation of adhesive mixtures for inhalation and mixing time is an easily controllable process parameter. Despite this, the effect of mixing time on formulation performance and its relation to all the other variables to be considered is still not fully understood for carrier-based inhalation formulations. As a start to straightening out this discrepancy this paper deals with the effect of mixing time on drug detachment for adhesive mixtures with a relatively low drug content. In particular interactions between mixing time and the type of drug, the carrier size fraction and the flow rate are investigated. Starting point of this study is the idea that the balance of mainly drug (de-) agglomeration, redistribution over and compression onto the carrier surface determines the overall effect of mixing time on drug detachment. Therefore, different characterisation techniques have been used in an attempt to measure or monitor the occurrence of these three ‘principal processes’ and, by that, to study their relative contribution to the overall effect.

## Materials and Methods

### Starting materials

Alpha lactose monohydrate (Pharmatose 80M, DMV-Fonterra Excipients, Goch, Germany) was used to prepare the different carrier size fractions. Micronised salmeterol xinafoate and fluticasone propionate were granted by Novartis (Germany). These drugs were chosen for their difference in cohesion-adhesion balance in combination with lactose, which was previously reported to be 2.39 and 0.22, respectively [20]. The drugs were passed through a 90 µm sieve to break up larger agglomerates and triboelectric charge resulting from the screening process was allowed to decay for at least 2 days afterwards.

### X-ray diffraction

The change in characteristics of drug particles when subjected to mechanical stress during mixing may depend on their initial solid state. For example, disordering or amorphisation of initially crystalline particles results in a higher apparent solubility [21,22]. Therefore, the solid state of the drugs as used was measured by X-ray diffraction with a D2 PHASER equipped with a 1 mm divergence slit and a LYNXEYE^TM^ detector (Bruker AXS B.V., Delft, The Netherlands). Approximately 15 mg of the micronised material was evenly spread on a zero background Si sample holder without the use of an adhesive. The sample holder was rotated at 60 rpm during measurement and it was made sure that the powder was still in place afterwards. An air-scatter screen of 1 mm was used to prevent radiation from the X-ray source to directly reach the detector. The scans were performed from 5 to 40 ° 2θ with a step size of 0.01 ° 2θ and a 1 s step duration. CuKα radiation with a wavelength of 1.5406 Å was generated at 30 kV and 10 mA. The measurements were performed on at least two different specimens from the same powder sample to ascertain the reproducibility of the obtained diffraction pattern.

### Carrier classification

A coarse (250-315 µm) and a fine (63-90 µm) carrier fraction were obtained from Pharmatose 80M by 20 minutes of vibratory sieving at an amplitude of 1.5 mm (Retsch AS 200 control, Germany). To further remove lactose fines from the surface of the carrier particles, the carrier fractions were then air jet sieved for 10 minutes (e200LS, Hosokawa Alpine AG, Augsburg, Germany); the coarse fraction on a 250 µm sieve at an underpressure of 2000 Pa and the fine fraction on a 63µm sieve at 3000 Pa.

### Blend preparation

Blends were prepared at ambient conditions. The coarse carrier fraction was blended with 0.4% of drug and the fine carrier with 1.48% to obtain a similar carrier surface payload (in mg/m^2^, calculated based on the ratio of the arithmetic mean fraction diameters). The drug was sandwiched in between two equal parts of the carrier material in a stainless steel mixing vessel with a volume of 160 cm^3^ and pre-mixed with a spatula for approximately 20 orbits. The blends were subsequently mixed with a Turbula blender operated at 90 rpm (WA Bachofen, Basel, Switzerland) for 0.5 to 780 minutes. Data for different mixing times are obtained from the same batch, starting with a batch size of 25 g which decreased to approximately 15 g for the final blending step due to the extraction of samples. For both carrier fractions placebo blends (containing only the carrier material) were prepared in the same way.

### Content uniformity testing

Content uniformity of the blends was tested by taking 10 samples of 25 ± 1 mg from random positions. Blend homogeneity was considered acceptable at relative standard deviations (RSDs) of the content < 3%. The mean value of the 10 samples was taken as the drug content in the mixture.

### Segregation sensitivity testing

To confirm a possible explanation for the content uniformity data, the segregation sensitivity of the salmeterol mixtures containing a coarse carrier and being mixed for 2 and 420 minutes was tested. To this end, 1 g samples were subjected to the described vibratory sieving procedure during 1 minute on a 150 µm test sieve. The salmeterol content of the sieving residue was determined from 5 samples of 25 ± 1 mg and expressed relative to the salmeterol content of the original blends.

### Scanning electron microscopy (SEM)

The (re-)distribution and agglomeration behaviour of the drug on the carrier with mixing time was studied with SEM. Images were obtained with a JSM-6301F (Jeol, Japan) at an acceleration voltage of 3kV and probe current 7. Samples were fixed on an aluminium specimen mount by means of double sided adhesive carbon tape. For the pure drugs excess sample was blown from the tape with pressurised air. Any excess particles from the carrier material and blends were gently tapped from the specimen mount to avoid detachment of lactose fines or drug from the carrier crystals, respectively. The drugs were sputter coated with 10 nm of a gold–palladium alloy, whereas for the carrier and mixture samples a coating thickness of 20 nm was found to be necessary for preventing charging effects.

### Laser diffraction analysis

All laser diffraction experiments have been performed with the HELOS BF diffractometer (Sympatec, Clausthal-Zellerfeld, Germany) equipped with an R3 lens (measuring range 0.9-175 µm) or an R5 lens (measuring range 4.5-875 µm, for the coarse carrier fraction). The FREE calculation mode was used, which is based on the Fraunhofer theory.

#### Dry dispersion

The particle size distributions (PSDs) of the drugs were measured after dispersion of the powders with a RODOS disperser at 3 bar (Sympatec, Clausthal-Zellerfeld, Germany). The PSDs did not change when the pressure drop for dispersion was increased to 5 bar, which indicates that the primary PSDs of the drugs were measured. Results are the mean of 2 measurements, which was deemed a sufficient number of replicates with the added control measurements at a dispersion pressure of 5 bar and considering the small deviations that were observed between individual measurements.

The PSDs of both lactose carrier fractions were measured in the same way at 3 and 5 bar. Approximately 2 g of the sieved and placebo blended material was fed to the RODOS disperser through a funnel. Results are the mean of 3 measurements.

#### Wet dispersion

To further quantify the qualitative information from SEM, the agglomeration behaviour of the drugs on the carrier with prolonged mixing was measured with laser diffraction too. The agglomerate size of the hydrophobic drugs as present in the screened starting material and in the blends was measured in aqueous suspensions using the CUVETTE SC-40 module (50 mL cuvette, Sympatec, Clausthal-Zellerfeld, Germany). A sample of the blend was added to saturated aqueous solutions of the drugs containing approximately 0.03% of polysorbate 80 (Tween 80). The particle size distributions of suspended drug agglomerates were measured for 10 s after precisely 12 minutes (coarse carrier) or 2 minutes (fine carrier); the duration in which the lactose completely dissolved for all samples. Dissolution of the lactose carrier was apparent from a disappearing peak corresponding to the size of the carrier material and further confirmed by optical microscopy of the suspensions (see further). A stirring speed of approximately 500 rpm was used throughout the entire procedure to prevent sedimentation of the suspended drug particles. It was made sure that the hydrophobic drugs did not adhere to the wall of the cuvette during measurement. Sample sizes were chosen such that an optical concentration of around 10% was obtained. It was checked that dissolved lactose did not influence the laser diffraction results. The primary PSDs of the drugs were measured after wet dispersion in a similar way after a pre-suspension step comprising sonication of approximately 0.5 mg of the drugs in 2 mL of the saturated solution for 11 minutes using a 70 W, 42 kHz ultrasonic cleaner (Electris UC449UP, France). The amount of pre-suspension that was added to the CUVETTE was titrated to an optical concentration of around 10%. It was checked that the optical concentration and characteristic PSD data of the primary particles thus measured remained constant for minimally 12 minutes. The chosen conditions and procedures are the result of their careful evaluation concerning reliability and reproducibility during many exploratory measurements. Results are the mean of at least 3 measurements.

### Optical microscopy

Aqueous suspensions of the adhesive mixtures were inspected by optical microscopy to confirm the dissolution of the lactose carrier (BX50F, Olympus Optical Co., Ltd., Japan). Several drops of the suspensions were placed on a glass microscope slide without using a cover slip.

### Solubility testing

The apparent solubility of salmeterol was measured as a means to quantify the degree of mechanical stress imparted on the drug particles during the mixing process. An aqueous suspension of salmeterol was prepared by suspending the micronised starting material in demineralised water containing approximately 0.03% polysorbate 80 (Tween 80). After sonication for at least 30 minutes in a Helma Transsonic 700/H ultrasonic bath (Elma Hans Schmidbauer, Singen, Germany) the suspension was stored in the dark for 1 week without stirring before further use. Thereafter, the suspension was passed through a 0.2 µm cellulose acetate filter to obtain a saturated solution. The apparent solubility strongly depends on the suspended drug concentration [21,22]. Therefore, to 10 mL of the saturated solution, 0.7 mg of the salmeterol starting material was added or a sample of the different salmeterol mixtures that resulted in an equivalent added salmeterol mass (calculated based on the measured content). The resulting suspensions were regularly vortexed during 1 hour. Exploratory measurements showed that, for the longest mixing times, maximum dissolution is attained within 1 hour and that the concentration consecutively decreases towards the equilibrium saturation concentration in the course of several days to weeks. The procedure was continued by passing the suspensions through a 0.2 µm cellulose acetate filter. The samples were further analysed as discussed in the section ‘spectrophotometric analysis’. Results are the mean of 2 measurements.

### Drug detachment experiments

Drug detachment was measured by analysing the residual amount of drug present on the carrier surface after a dispersion experiment with a classifier based test inhaler [23]. The carrier crystals could be collected for analysis after a drug detachment experiment, since they were retained in the classifier of the inhaler. The residual amount of drug normalised to 100% of the carrier is referred to as ‘carrier residue’ (CR). The percentage of drug detached is calculated as 100-CR. Doses of 25 ± 1 mg were used for the drug detachment experiments, which were performed at flow rates of 20 and 60 L/min for a fixed duration of 3 seconds. Drug detachment experiments for the same flow rate and drug-carrier combination (at different mixing times) were performed on the same day to minimise environmental effects. Results are the mean of 5 measurements.

### Spectrophotometric analysis

Samples from the content uniformity analyses and drug detachment experiments were analysed for salmeterol and fluticasone content by spectrophotometric analysis at a wavelength of 228 nm (Unicam UV-500, ThermoSpectronic, Cambridge, UK). Calibration curves for the concentration of both drugs were constructed with a coefficient of determination of 0.9998. All samples were dissolved in ethanol and subsequently centrifuged for 5 minutes at 3000 rpm (Hettich Rotanta D-7200, Hettich AG, Switzerland) to clear the drug solutions from suspended lactose particles prior to measurement. If necessary, samples were diluted for the drug concentrations to fall within the range covered by the calibration curves.

The absorbance of the samples from the solubility tests was measured spectrofotometrically at a wavelength of 280 nm. The measurement at this wavelength avoids the necessity of dilution of the supersaturated salmeterol solutions in order for the absorption values to fall within the linear measuring range of the spectrophotometer used. Water containing 0.03% of polysorbate 80 and the dissolved pure lactose carrier (if applicable) was used as a blank. Because only the relative difference in the apparent solubility of salmeterol between the different samples is of interest, an exact quantification of the salmeterol concentration is not necessary and no calibration curve was constructed.

## Results and Discussion

### Solid state of the drugs

**Figure 1:**
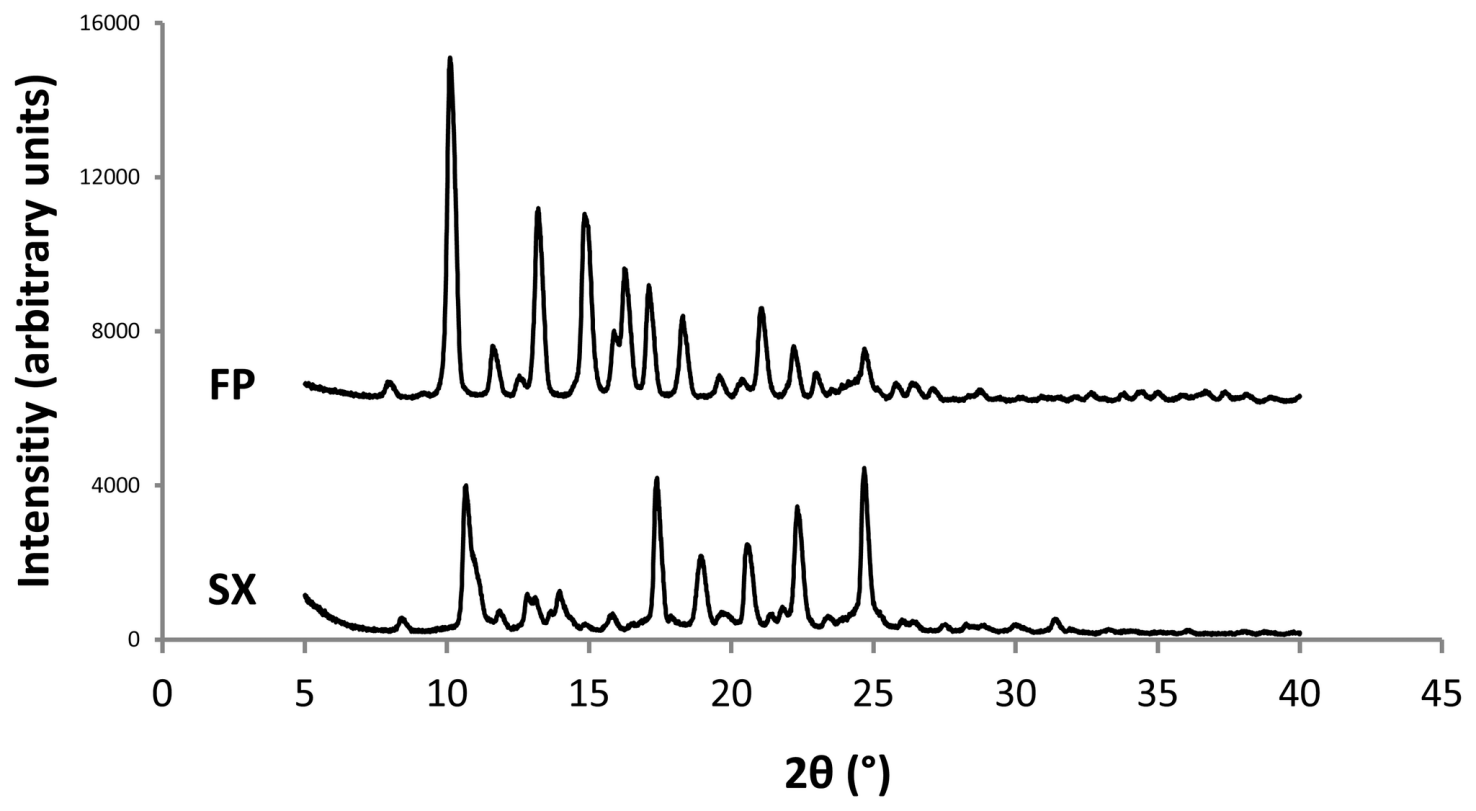
X-ray diffraction data for salmeterol xinafoate (SX) and fluticasone propionate (FP).

The X-ray diffraction patterns of the drugs are presented in Figure 1. Judging from the sharp peaks and the lack of a ‘halo’ both drugs are crystalline.

### Particle size distributions of the drugs and carrier materials

Because the volume median diameters of the drugs are well within the desirable size range for inhalation of 1-3 µm (see Table 1), the drugs are considered suitable for use in these experiments.

**Table 1:** Characteristic PSD data (average (SD)) of the drugs (n = 2) and carrier material (n = 3).

	**X50 (µm)**	**V% < 10 µm**
Salmeterol (dry)	1.41 (0.01)	99.99 (0.01)
Salmeterol (wet)	1.43 (0.01)	100
Fluticasone (dry)	1.80 (0.06)	99.85 (0.03)
Fluticasone (wet)	1.59 (0.02)	99.53 (0.04)
Fine carrier	72.04 (0.28)	1.65 (0.06)
Fine placebo*	74.75 (0.60)	1.15 (0.51)
Coarse carrier	289.97 (4.29)	0.34 (0.41)
Coarse placebo*	288.69 (0.87)	0.15 (0.01)

* The fine and coarse carrier as obtained after the sieving procedure have been placebo blended for 600 and 420 minutes, respectively.

**Figure 2:**
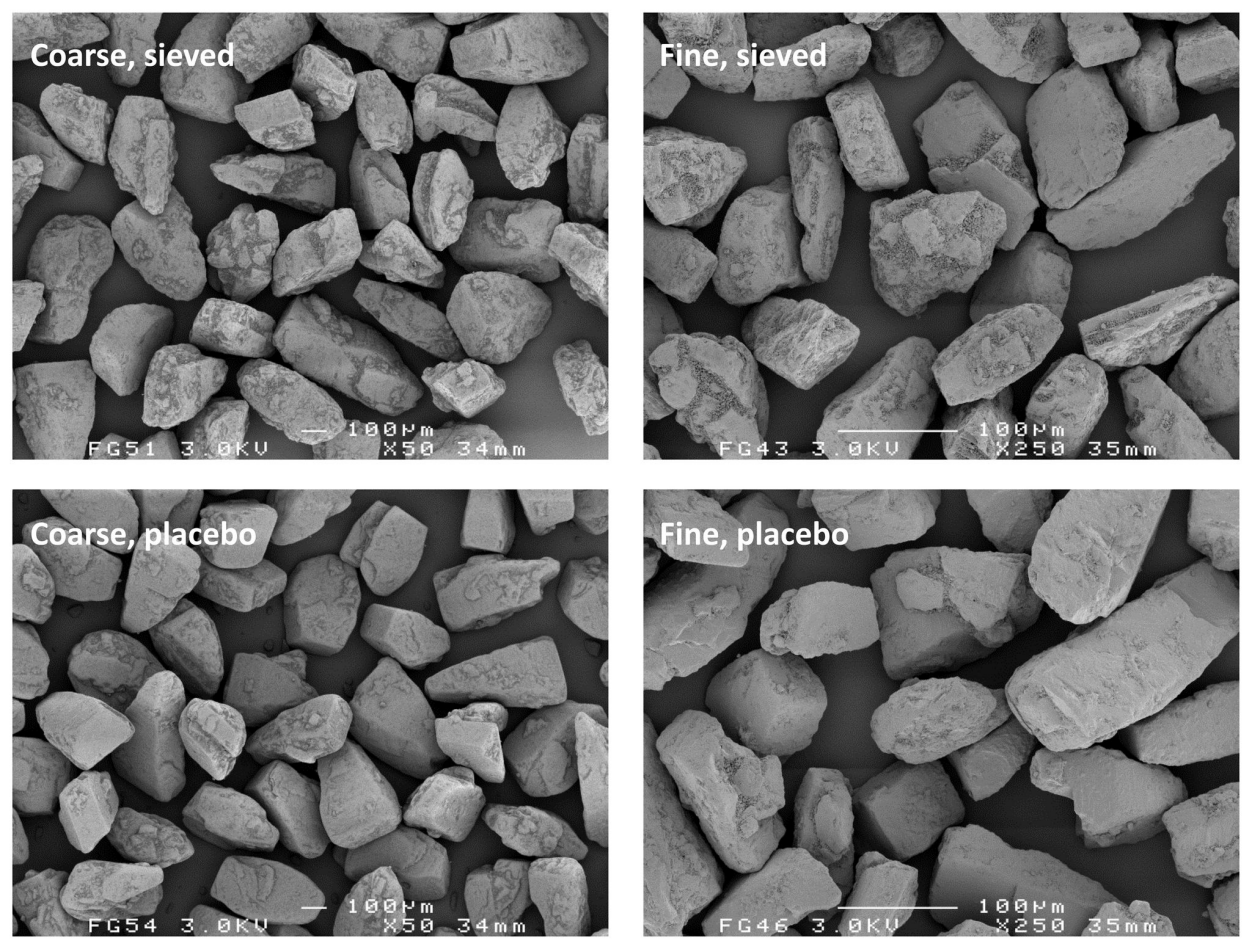
Representative SEM images of the sieved and placebo blended carrier material. Mixing times for the placebo blends displayed are 420 and 600 minutes for the coarse and fine fraction, respectively.

The carrier material as obtained after the classification procedure still contained a measurable amount of fines, which is presented in Table 1 as the volume fraction < 10 µm. Placebo mixing of both carrier fractions unexpectedly resulted in a trend of decreasing volume fraction < 10 µm. The loss of fines with mixing was confirmed by visual observation of the carrier particles with SEM (Figure 2). The reduction of the amount of fines may be explained by their adhesion to the inner walls of the mixing vessel, which resulted in a faint white haze being visible after placebo mixing.

The data from RODOS dispersion suggest that the fine carrier fraction contains a higher volume fraction of lactose fines than the coarse carrier fraction. However, because the specific surface area of the fine carrier fraction is approximately 3.7 times that of the coarse carrier (calculated based on the ratio of the arithmetic mean fraction diameters), their fines content per unit carrier surface area may well be comparable. This statement is supported by Figure 2, in which both carrier fractions are shown at a different magnification so as to display the carrier particles at the approximate same size. There is no notable difference in surface coverage by fines between the fine and coarse carrier fractions. Differences in the size distribution of the lactose fines may exist between the carrier fractions, however.

The appearance of lactose fines in commercial carrier products is inevitable as they cannot be effectively removed. They will influence the balance of the principal processes (i.e. drug (de-) agglomeration, redistribution and compression) during mixing of the blends to certain extent. Based on the placebo experiments it may be expected that in our study drug detachment will not be affected by mixing time through the generation of new lactose fines. Furthermore, any difference in the effect of mixing time on drug detachment between both carrier fractions is not likely to be the result of a difference in the covering of the carrier by lactose fines. However, it is not clear to what extent this may be determined by other factors too, such as differences in the scale of carrier surface discontinuities between the carrier fractions.

### Content uniformity

A trend of decreasing drug content with prolonged mixing was observed for both drugs mixed with the coarse carrier (Table 2, 0.4% of drug). This is the result of drug adhesion to the inside of the mixing vessel, which was observed as the formation of a white haze that became more opaque as mixing was continued. For the fine carrier, drug losses occurred mostly at the early onset of mixing after which the content stayed relatively constant (Table 2, 1.48% of drug). The difference in absolute drug loss to the mixing vessel wall between the carrier fractions is small (approximately 15 and 18.5 mg for the coarse and fine carrier fraction, respectively), which suggests that the drug loss is largely determined by saturation of the mixing vessel’s inner walls, which is independent of the carrier type. The losses are of the same order of magnitude for both drugs.

**Table 2:** Content uniformity test results for blends containing 0.4% drug on a coarse lactose carrier or 1.48% drug on a fine lactose carrier (n = 10).

	**0.4% salmeterol**		**0.4% fluticasone**		**1.48% salmeterol**		**1.48% fluticasone**	
**Mixing time**	Content*	**RSD (%)**	Content*	**RSD (%)**	Content*	**RSD (%)**	Content*	**RSD (%)**
0.5 min	95.3	0.91	97.4	1	95.5	0.96	96.5	1.23
2 min	94.9	0.89	97.1	0.8	96.5	0.75	95	1.47
10 min	93.5	0.61	94	0.82	96.7	0.37	95.7	0.53
30 min	90.3	0.56	90.7	0.56	96.3	0.86	94.5	0.68
60 min	87.3	0.92	89.9	0.51	96.4	0.49	94.4	0.88
120 min	76.5	0.82	86.1	0.73	96.2	0.72	93.9	0.88
420 min	84.9	2.54	84.2	1.16	94	0.89	92.6	0.34
600 min	93.4	0.82	94.7	0.41				
780 min	94.6	0.49						

* Content = % of drug weighed.

For all blends the RSD of the content is < 3% and they are therefore considered homogeneous (Table 2). No meaningful change in RSD with increased mixing time is observed, only the RSD of the 0.4% salmeterol mixture after 420 minutes of mixing is notably higher. This higher RSD accompanies an increase in segregation sensitivity. Between 2 and 420 minutes of mixing the drug loss resulting from 1 minute of vibratory sieving increases from 15.6% to 52.4%.

### Agglomeration effects

#### Scanning electron microscopy

**Figure 3:**
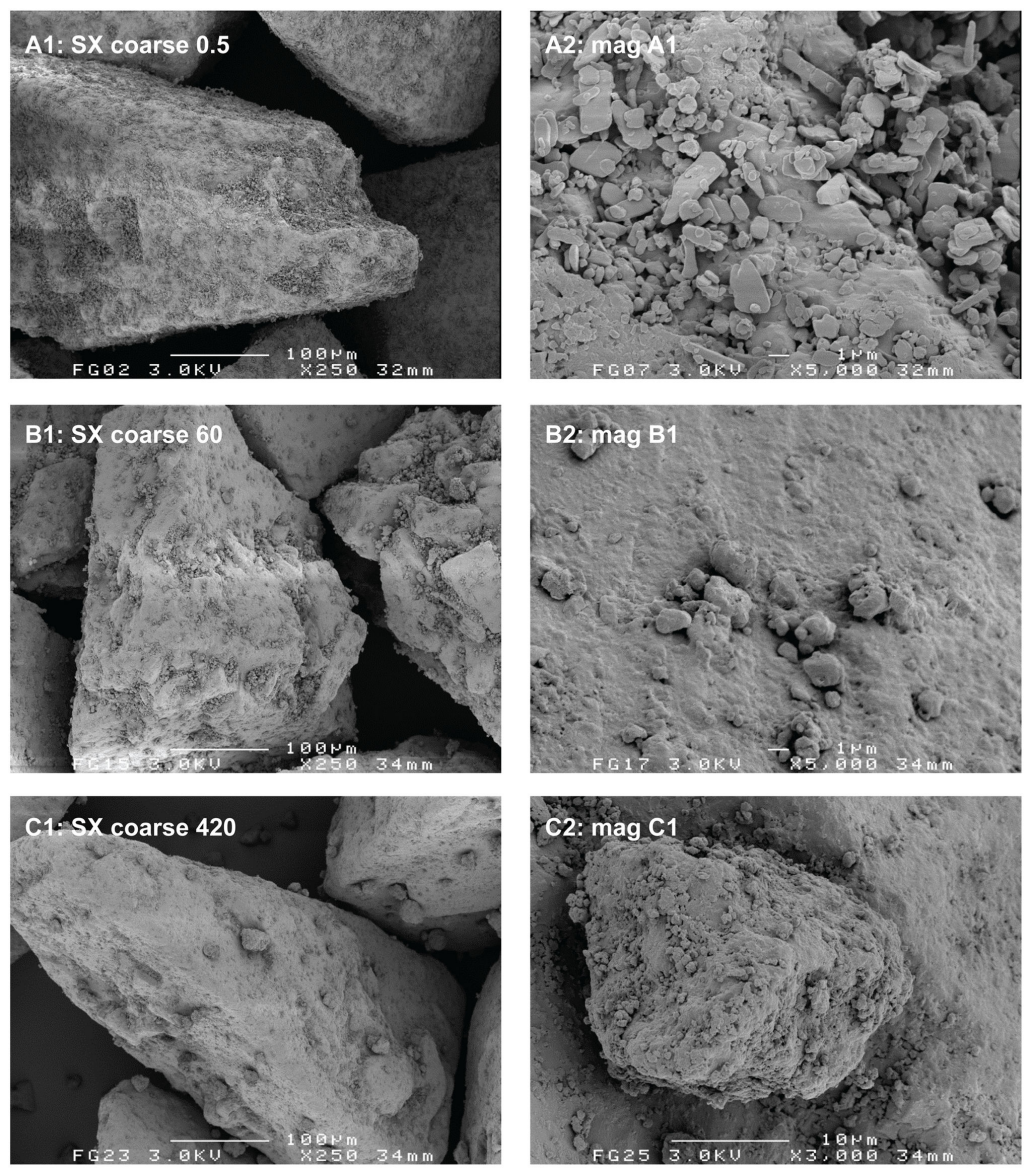
Representative SEM images of the salmeterol blend containing a coarse carrier at different mixing times. Mixing times are given in minutes. Magnifications on the right hand side are taken from the images on the left hand side.

**Figure 4:**
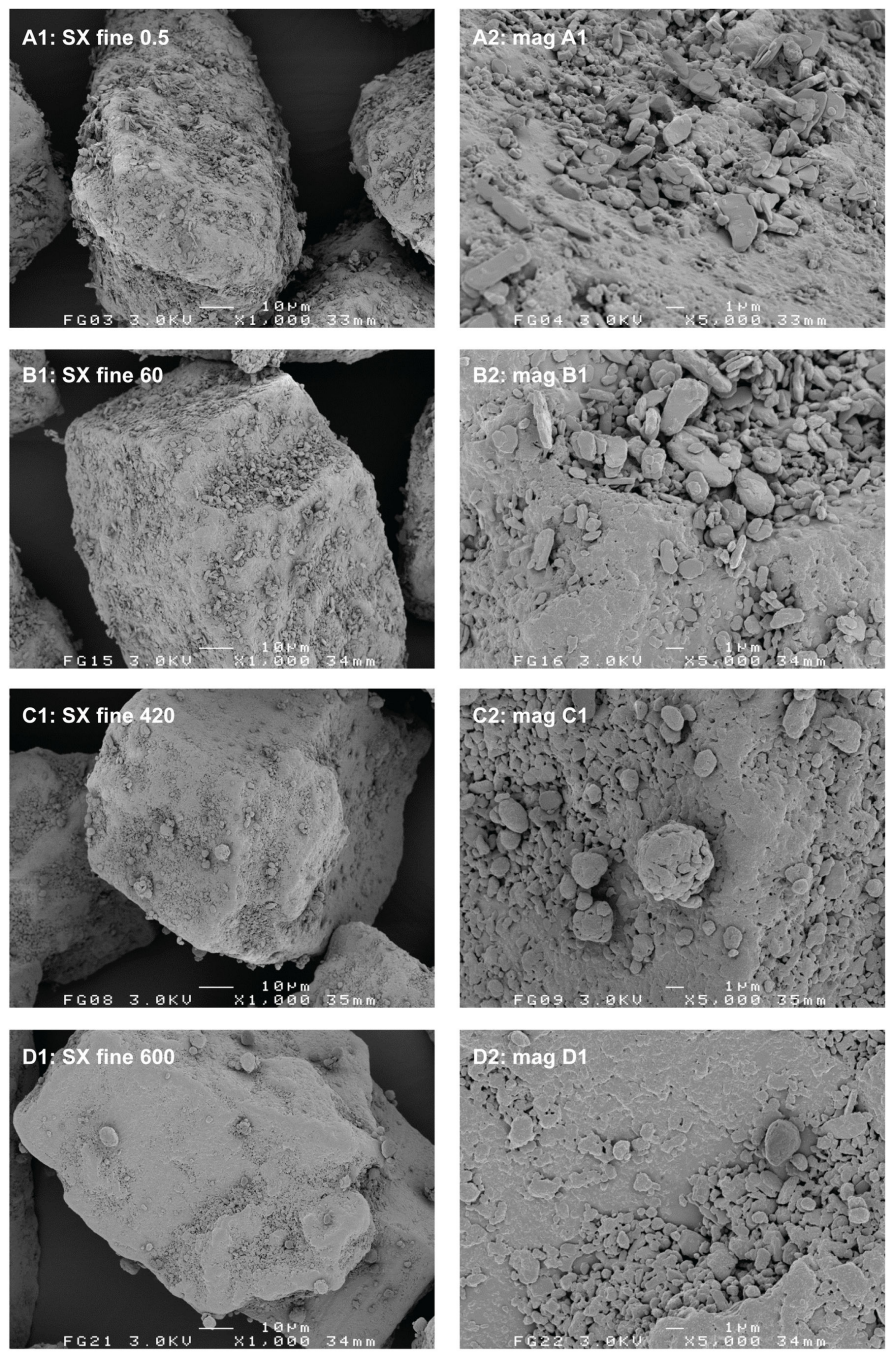
Representative SEM images of the salmeterol blend containing a fine carrier at different mixing times. Mixing times are given in minutes. Magnifications on the right hand side are taken from the images on the left hand side.

The relatively lower degree of homogeneity and increased segregation sensitivity for the 420 min 0.4% salmeterol mixture are likely the result of a high degree of agglomeration of the salmeterol (and possibly fine lactose) particles onto or in between the coarse carrier particles. For the large agglomerates of approximately 25 µm that are visible in Figure 3 (C1 and C2) gravitational forces start to dominate adhesion forces. This shifts the balance between randomisation and ‘ordering’ or adhesion in favour of randomisation [4]. The reproducibility of the formation of large agglomerates was confirmed by SEM for at least three different batches of 0.4% salmeterol prepared. A less pronounced agglomeration of salmeterol with prolonged mixing is observed on the fine carrier (Figure 4). The agglomerates are less numerous and they are smaller than those on the coarse carrier (no larger agglomerates than about 10 µm could be found in the specimen). This observation suggests that agglomeration may occur in the carrier surface irregularities, which are larger for the coarse carrier. These data are in line with the conclusion from previous studies that the size of drug agglomerates after mixing increases with increasing carrier particle size [7,17]. However, the conclusions from those studies are based on mixtures containing equal drug contents. This results in a higher carrier surface payload (mg/m^2^) for larger carrier size fractions, which may also cause an increase in agglomerate size. In our study a difference in carrier surface payload cannot have caused the difference in agglomeration behaviour between both carrier size fractions, as it was kept constant.

**Figure 5:**
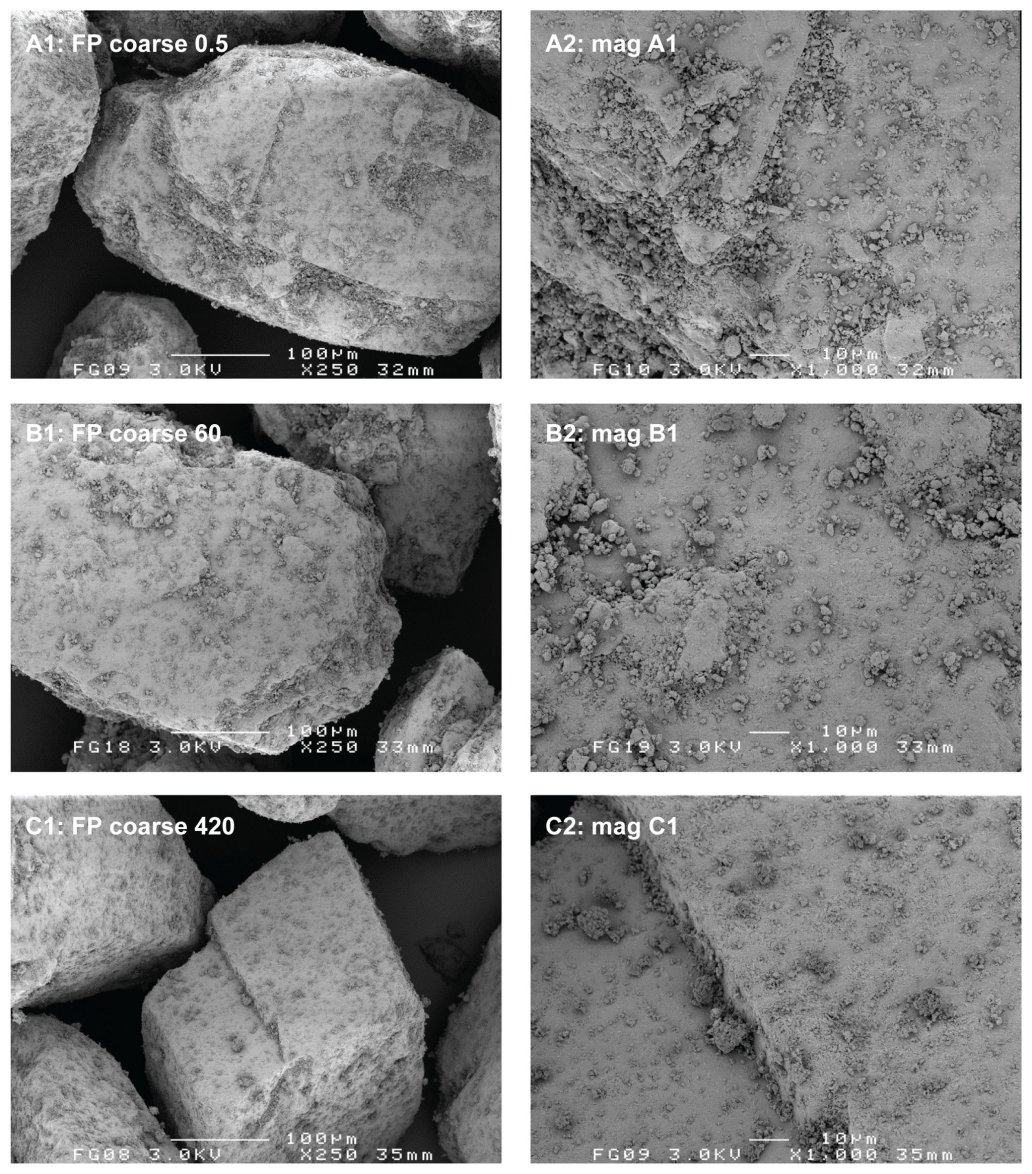
Representative SEM images of the fluticasone blend containing a coarse carrier at different mixing times. Mixing times are given in minutes. Magnifications on the right hand side are taken from the images on the left hand side.

**Figure 6:**
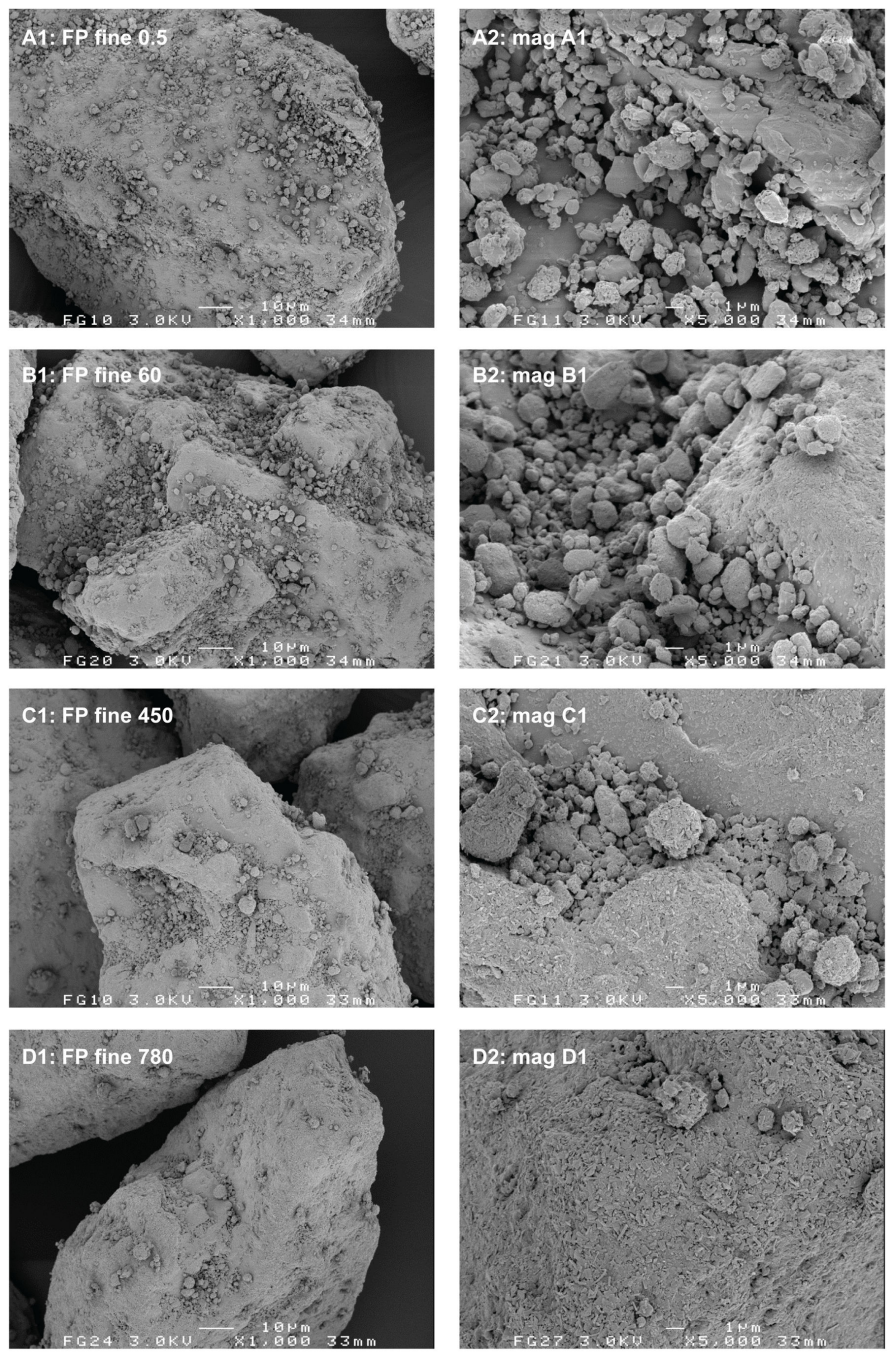
Representative SEM images of the fluticasone blend containing a fine carrier at different mixing times. Mixing times are given in minutes. Magnifications on the right hand side are taken from the images on the left hand side.

The agglomeration behaviour of fluticasone is markedly different from that of salmeterol when mixed with the coarse carrier depending on the mixing time. Some fluticasone agglomerates could be found in the specimen after 420 minutes of mixing (Figure 5, C1 and C2), but they are smaller (maximally about 10 µm) and less numerous than is the case for salmeterol (Figure 3, C1 and C2). When mixed with the fine carrier, the difference in agglomeration behaviour between fluticasone (Figure 6) and salmeterol (Figure 4) is not as pronounced. Evaluations based on SEM images can lead to biased or incorrect conclusions, however. Disadvantages inherently associated with SEM imaging include the difficulty of representative sampling from the mixture, representative imaging of a specimen and the possible altering of the sample during its preparation. SEM images presented in this paper are therefore images that have been obtained with utmost care to ascertain their representativeness. Their selection has been made after studying multiple samples and batches and imaging different spots of the same specimen. In addition, care was taken for the gentle handling of the powder during sample preparation. Nevertheless, one should keep in mind that conclusions from SEM imaging are based on a very limited number of observations, which is why laser diffraction was used in this study as an additional characterisation technique.

The difference in the propensity towards agglomeration between both drugs, which becomes especially notable after prolonged mixing with the coarse carrier, can be explained by a difference in their balance of intrinsic cohesive to adhesive interaction energy in combination with lactose. Values for the cohesion-adhesion balance (CAB) of salmeterol and fluticasone with lactose have previously been reported to be 2.39 and 0.22, respectively [20]. This means that the cohesiveness of salmeterol is 2.39 times its adhesiveness to lactose, whereas for fluticasone the adhesiveness to lactose is 4.55 times its cohesiveness. Although these results have been obtained with different batches of material (which may influence CAB values significantly [24]), they are in agreement with the greater agglomeration tendency of salmeterol than that of fluticasone observed in this study.

#### Laser diffraction analysis

**Figure 7:**
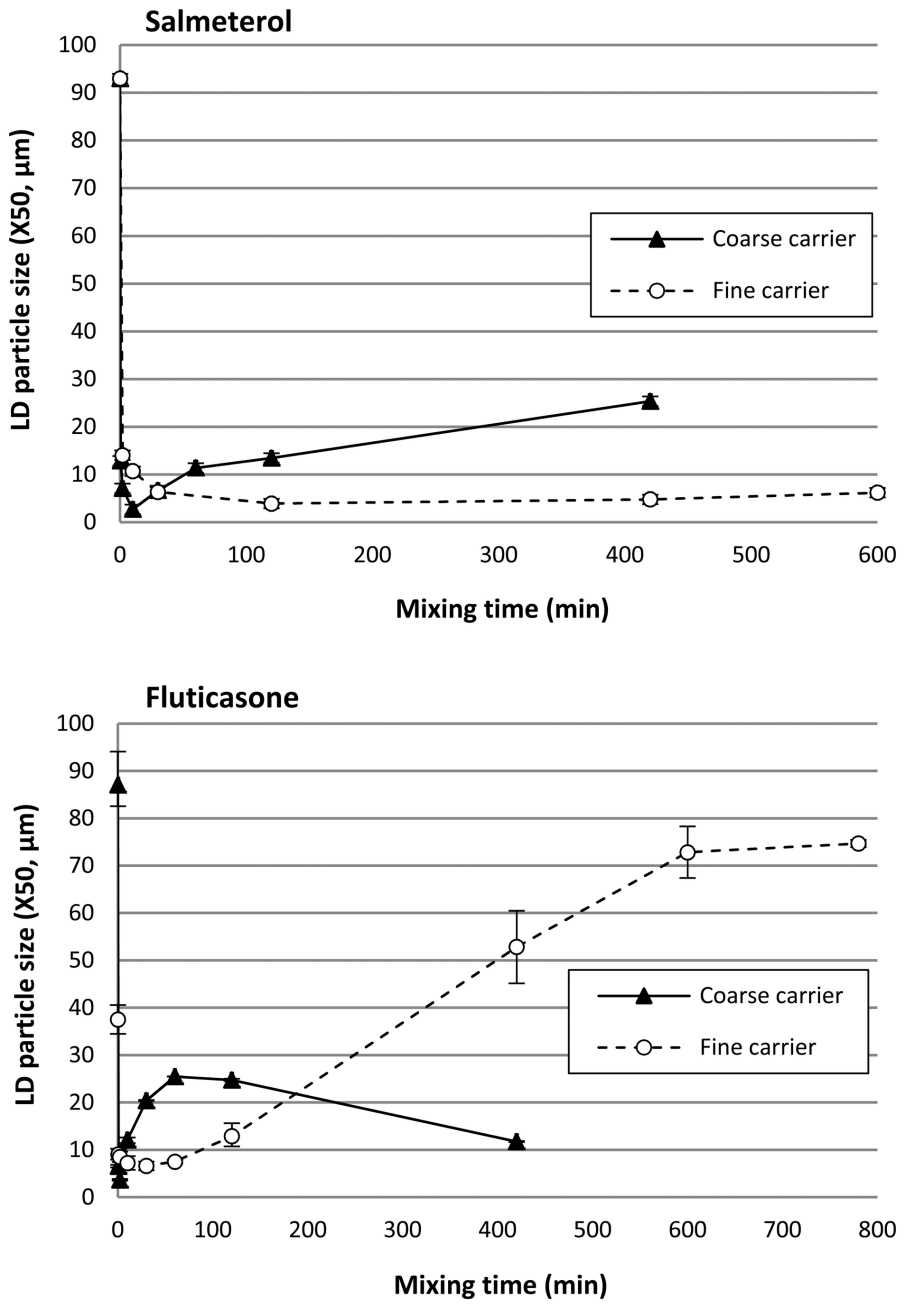
X_50_ from laser diffraction analysis of suspended drug particles after dissolution of the carrier material. Y-error bars represent minimum and maximum values measured (n = 2).

The wet suspension laser diffraction method provides data on the agglomeration behaviour of salmeterol that are in agreement with the observations from SEM (Figure 7). The X_50_ of 93 µm at t = 0 min represents the agglomerate size of the screened starting material. These agglomerates are quickly dispersed during blending with the coarse carrier until a minimum X_50_ of only 2.71 µm is reached after 10 minutes, which approaches the primary particle size of the drug (Table 1). Continued mixing then results in a gradual increase of the X_50_ to a value of 25.4 µm after 420 minutes. As discussed, agglomerates of the same order of magnitude have been observed with SEM (Figure 3, C1 and C2). For the fine carrier fraction, a minimum in X_50_ is reached after 120 minutes, which remains very much the same (around 5 µm) during continued mixing. Compared to the X_50_-value of the primary salmeterol particles (Table 1) this confirms the occurrence of only minor agglomeration, as was concluded from SEM micrographs too.

**Figure 8:**
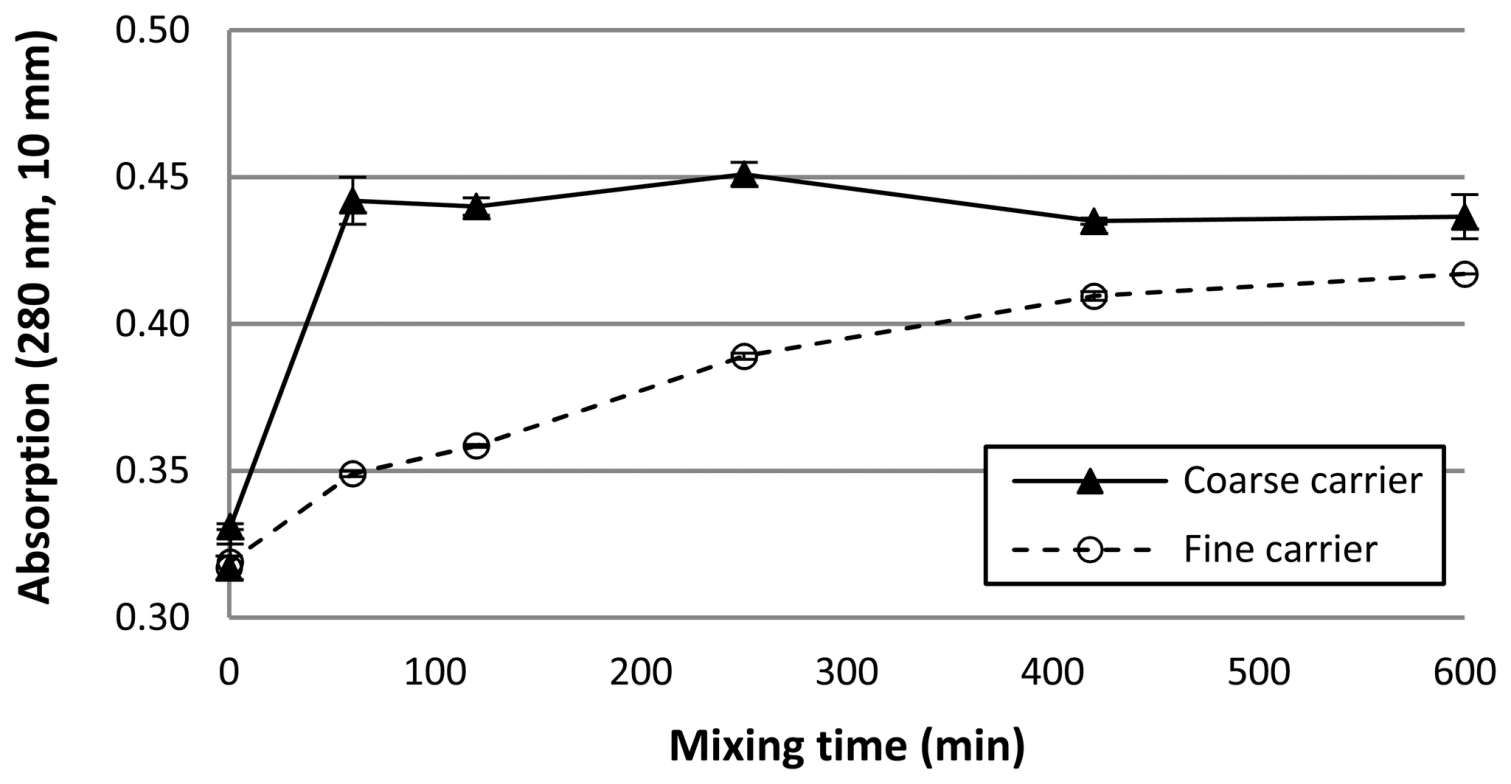
Change in apparent solubility of salmeterol during mixing with the coarse and fine lactose carrier. The apparent solubility is represented by the absorption of the solution at 280 nm.

Although these data for salmeterol may be in agreement with the images from SEM, they are likely to be biased by dissolution effects and subsequent deagglomeration in suspension. It was noticed that with prolonged mixing a larger sample of the blends was required to attain the desired optical concentration of around 10% (up to a 6-fold difference between the shortest and longest mixing times for both carrier fractions). This observation can neither be explained by the slightly lower drug content with prolonged mixing (Table 2), nor by differences in the particle size distribution of the agglomerates (especially for the fine carrier). It is much more likely the result of an increasing apparent solubility of salmeterol with increased mixing time that causes improved dissolution of the drug and thus the formation of a supersaturated solution (Figure 8). Therefore, the conclusion has to be drawn that the salmeterol data in Figure 7 may to certain extent be biased by dissolution effects, especially for long mixing times. This may result in enhanced dispersion of agglomerates, and thus underestimation of the agglomerate size.

**Figure 9:**
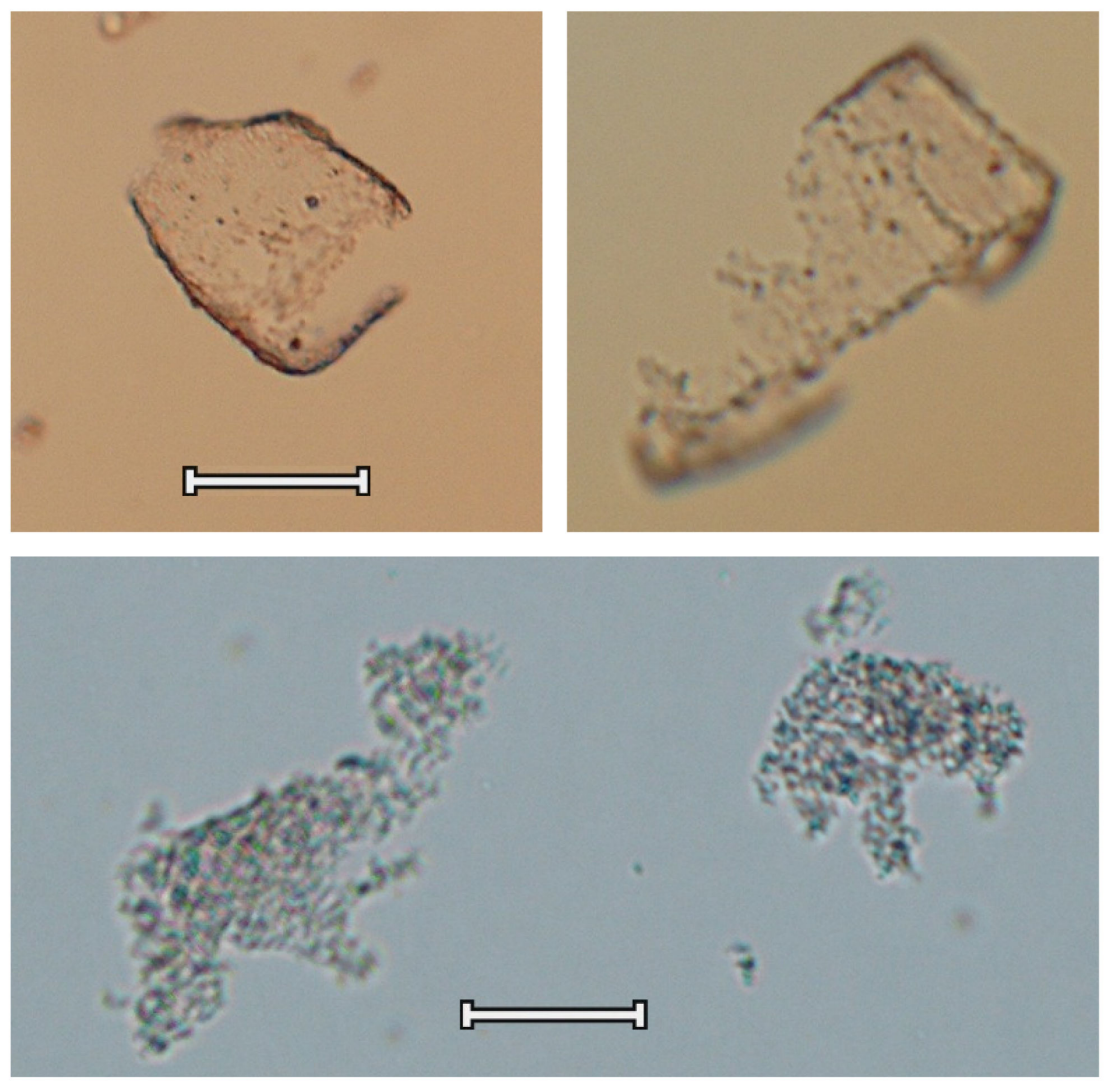
Light microscopic images of suspended fluticasone films after dissolution of the lactose carrier. Top: 1.48% fluticasone mixed with the fine carrier for 600 minutes, the scale bar represents 60 µm; bottom: 0.4% fluticasone mixed with the coarse carrier for 420 minutes, the scale bar represents 15 µm.

For fluticasone, laser diffraction data are not in agreement with the SEM images (Figure 7). With the coarse carrier, a maximum agglomerate size in the mixture of 25 µm is measured after 60 minutes of mixing, but agglomerates of this size could not be observed in the mixture by SEM (Figure 5, B1 and B2). For the fine carrier the difference is even more pronounced with a maximum of 75 µm after 780 minutes of mixing determined by laser diffraction measurements, whereas only small agglomerates of maximally 4-10 µm could be found with SEM (Figure 6, D1 + D2). The value of 75 µm coincides remarkably well with the size of the carrier particles. Light microscopic imaging of the suspensions revealed that this discrepancy is the result of the formation of insoluble, thin films that consist of the drug material and have sufficient structural integrity to remain intact in suspension after complete dissolution of the carrier. For the fine carrier these films have shapes similar to those of the carrier particles (Figure 9, top), whereas for the coarse carrier the films cover a relatively smaller part of the particle surface (Figure 9, bottom). Possibly the larger surface irregularities prevent the formation of a continuous film over the complete surface of the coarse carrier. Spontaneous re-agglomeration of drug particles after suspension is a well-known possible source of bias with wet laser diffraction measurements too. However, such a process is not likely to have contributed to the discrepancy between results from laser diffraction and SEM for fluticasone: no increase in the X_10_, X_50_ or X_90_ values was observed for any of the mixture samples during or after dissolution of the carrier material or for the suspended primary particles.

The formation of a film or coating on the carrier surface by both drugs can be observed with SEM too, especially on the fine carrier (Figures 4 and 6, D2). For salmeterol, film formation on the fine carrier could also be confirmed by suspension of the blend mixed for 600 minutes in a supersaturated drug solution. Then, similar films as shown in Figure 9 (top) for fluticasone were observed by light microscopic imaging several minutes after submersion of the particles (images not shown). This confirms that dissolution and dispersion prior to the laser diffraction measurements (Figure 7) must have occurred for salmeterol. The apparent solubility of fluticasone in the medium used in this study is too low to be reliably measured by spectroscopy, even after prolonged mixing. This explains why dissolution effects did not occur for fluticasone and the films remained intact in the suspension medium during the laser diffraction measurements.

Dissolving the carrier material to allow the measurement of drug agglomerates in suspension is a commonly applied technique [5,6,17]. However, such data are a measure of the strength of agglomerates as well as their size, because de-agglomeration can occur during the time required for dissolution of the carrier. This will be more pronounced for weaker agglomerates. In addition, fines of the same material as the carrier will be dissolved, which may cause the dispersion of composite agglomerates. Our results furthermore show that changes in the apparent solubility of the drugs and film formation may introduce additional sources of bias. With the mixing conditions applied in this study this is especially true for mixing durations longer than 1 hour. Therefore, the data from such wet laser diffraction methods have to be interpreted with good knowledge of all the processes involved and cannot be used unconditionally as a measure for the size of detachable drug agglomerates. To aid in their correct interpretation, supporting data from other characterisation techniques should preferably be provided.

### Press-on effects

The increase in apparent solubility of salmeterol (Figure 8) can be explained by the mechanical stress that is imparted on the drug particles during mixing. This causes disordering or amorphisation of the initially crystalline particle surface [25,26]. Mixing with the coarse carrier results in a faster increase in apparent solubility than does mixing with the fine carrier. Apparently, the higher mass of the coarse carrier particles results in increased mechanical stress on the drug particles during collisions and this leads to faster amorphisation. Within 60 minutes of mixing a plateau is reached in the apparent solubility which is roughly 1.5 times the supposed equilibrium solubility of the starting material. This means that a maximum degree of disordering of the salmeterol particles is reached within this time or that the suspension concentration chosen in these experiments is not optimal for distinguishing further disordering [26].

**Figure 10:**
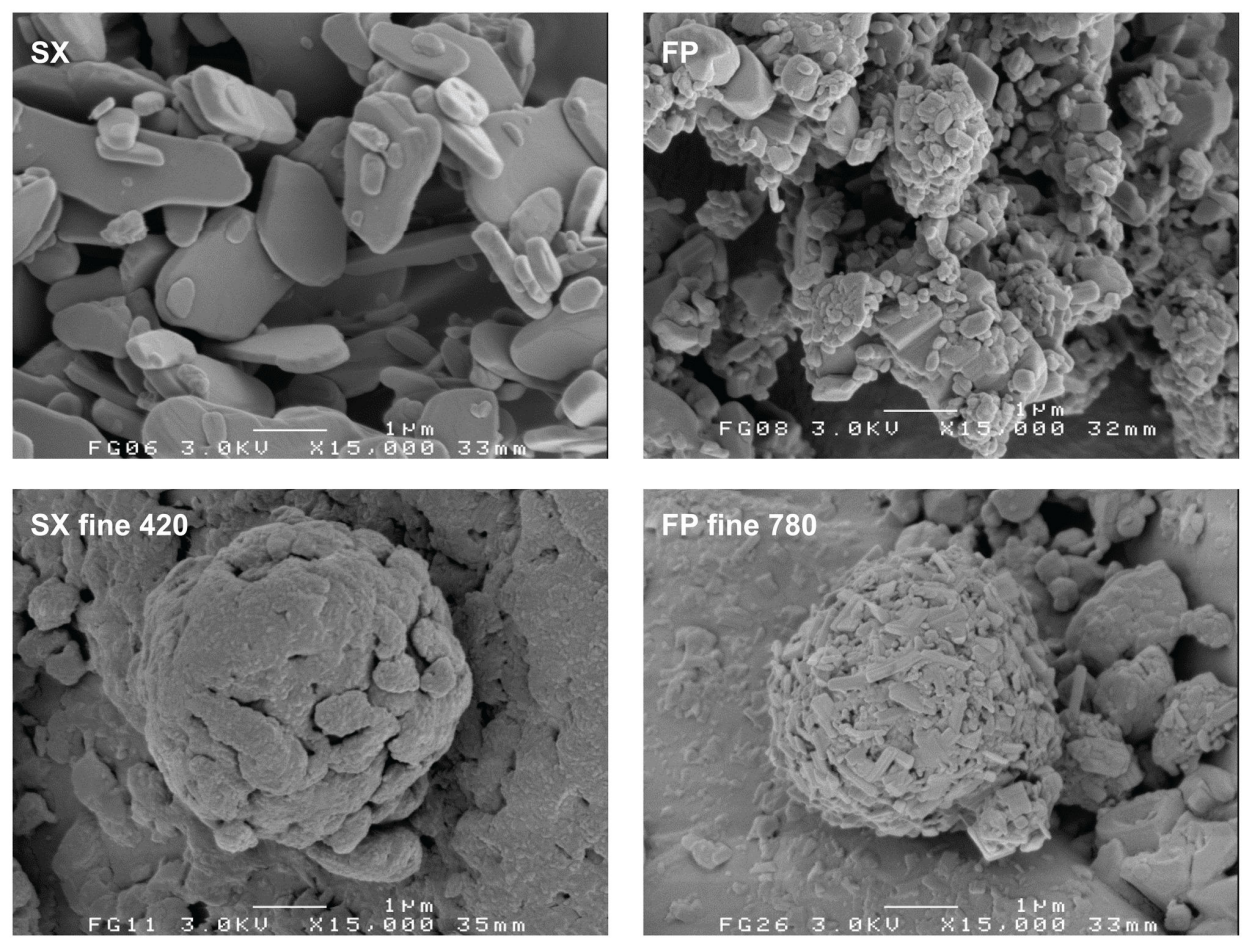
Representative SEM images showing morphological changes to the drug particles occurring during mixing. Top: the salmeterol (SX) and fluticasone (FP) starting materials; bottom: drug agglomerates on the fine carrier after a certain mixing time (in minutes).

The fact that the drug particles are subjected to significant mechanical stress during mixing does not only become clear from the increase in apparent solubility of salmeterol. SEM imaging of the blends reveals a change in morphology of the individual drug particles upon prolonged mixing too. This is most clearly illustrated in Figure 10, where the starting material is compared with agglomerates found in the mixture with a fine carrier after 420 (salmeterol) or 780 minutes (fluticasone) of mixing. Similar agglomerates and similarly shaped primary particles were not observed in the placebo blends. Therefore, the agglomerates shown in Figure 10 are likely to consist primarily of the drug component. The original plate-like salmeterol particles are extensively plastically deformed, whereas fluticasone appears to be fragmented into small needle shaped particles. In the mixture with the coarse carrier, plate-like salmeterol particles have mostly been deformed to spherical particles already after 60 minutes of mixing (Figure 3, B2). When mixed with the fine carrier, the original plate-like shape of the salmeterol particles is still recognisable after 60 minutes of mixing (Figure 4, B2). Only after 420 minutes of mixing their original shape is completely lost (Figure 4, C2). Therefore, the conclusion from the apparent solubility data that greater mechanical stress is caused by the coarser carrier is in agreement with the SEM images. Apparently, the larger carrier surface irregularities of the coarser carrier particles do not offer sufficient protection to the drug particles from mechanical stress to balance the higher frictional and inertial forces that result from a higher mass of this carrier.

The introduction of a higher degree of solid-state disorder with prolonged mixing may affect the chemical stability of the formulation. In addition, recrystallisation at the drug-carrier interface during storage may severely affect the powder’s dispersion behaviour in a negative way. The inertial and frictional mixing forces that cause an increase in apparent solubility may partly act as press-on forces, which cause increased drug-carrier interactions and thus negatively influence drug detachment during inhalation. Based on the faster increase in apparent solubility (Figure 8) a greater negative contribution from press-on forces to the overall effect of mixing time on drug detachment may be expected for the coarse carrier than for the fine carrier.

### Drug (re-)distribution

Drug particles are primarily located in carrier surface irregularities after 0.5 minutes of mixing (Figures 3-6, A1 and A2). The presented data indicate that the drug particles are subsequently redistributed over the complete carrier surface during prolonged mixing, where they may become immobilised by compression on the carrier surface to form continuous, coherent films. In addition, certainly for salmeterol, a substantial fraction of the drug forms large agglomerates (whether preceded by drug redistribution into carrier surface irregularities or not). Agglomeration and compression (as a result of mechanical stress that concurrently causes the film formation and increased solubility) of the drug particles are especially pronounced after mixing for at least 60 minutes. Therefore, after such long mixing times, the redistribution of drug particles between carrier surface sites with a different intrinsic binding activity is likely to be of minor importance than press-on and agglomeration effects to the overall effect of mixing time on drug detachment. With shorter mixing distribution effects may be more significant.

### Drug detachment

**Figure 11:**
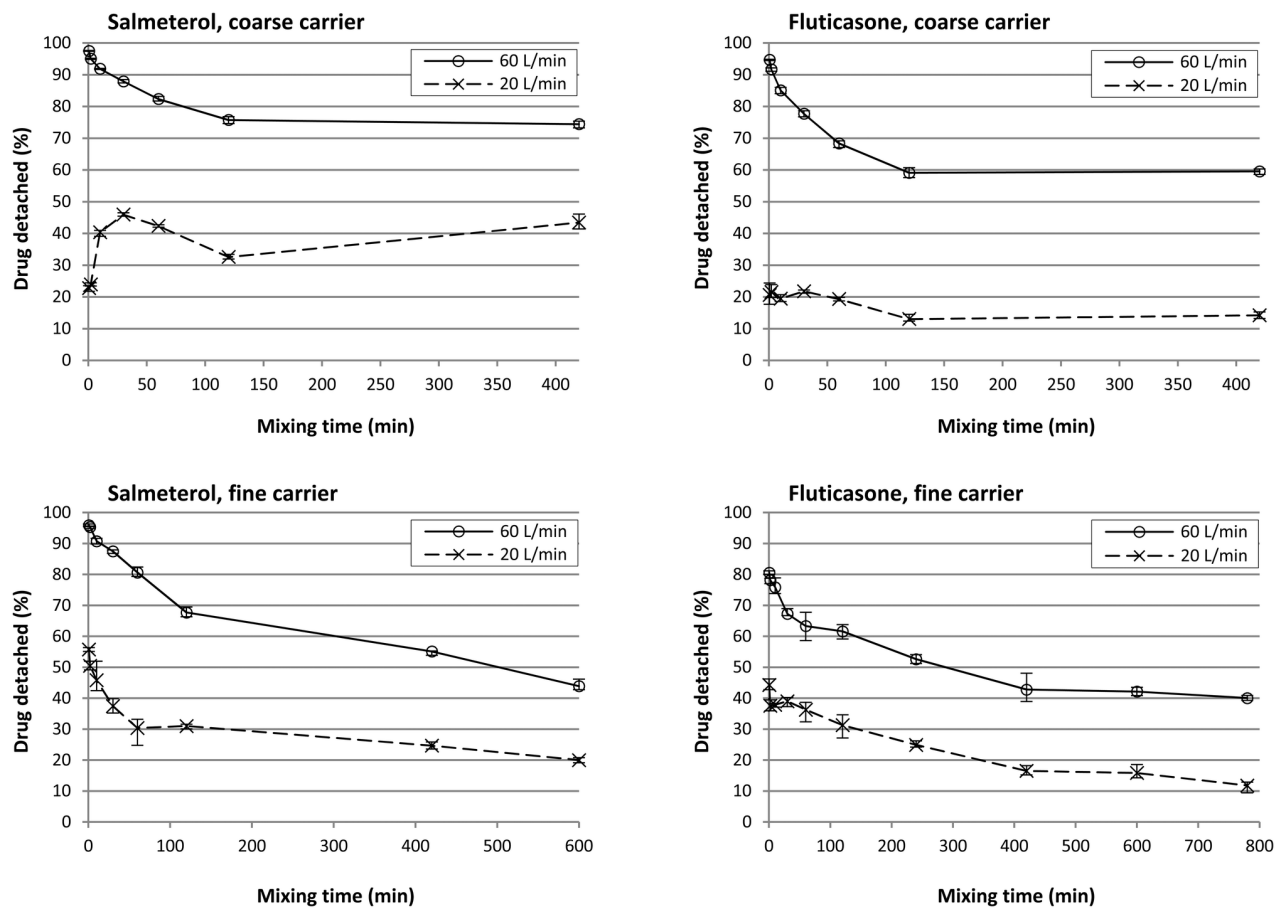
Drug detachment at 20 and 60 L/min as function of mixing time. Y-error bars represent minimum and maximum values measured (n = 5).

The data presented in Figure 11 confirm that the effect of mixing time on drug detachment is dependent on the type of drug, the carrier size fraction and the flow rate. The effect of mixing time is most pronounced within the first 120 minutes of mixing.

For salmeterol mixed with the coarse carrier, drug detachment at a flow rate of 20 L/min increases from 23.9% after 2 minutes of mixing to 43.4% after 420 minutes of mixing. These values are in line with the change in the amount of drug that is contained in easily detachable agglomerates, as was measured with the segregation sensitivity test (i.e. 15.6% to 52.4%, respectively). Therefore, the positive effect may be the result of a dominance of agglomerate formation. However, the maximum increase in drug detachment is already attained within the first 30 minutes of mixing, whereas agglomeration seemed to occur mostly from 10 to 420 minutes of mixing (based on the laser diffraction data in Figure 7) or from 60–420 minutes of mixing (based on the SEM images in Figure 3). Therefore, other effects may have contributed to the positive effect as well. The observed change in the shape of the salmeterol particles by SEM could be one of those additional effects (as shown in Figure 3, B2). At 60 L/min, the effect of mixing time is dominated by press-on effects and possibly some migration of drug particles towards active binding sites (distribution effects). In the first 10 minutes of mixing deagglomeration may also contribute to a lower drug detachment (i.e. a lower ratio of inertial separation to binding force due to a lower drug agglomerate mass). As a result, drug detachment decreases from nearly 100% after 0.5 minutes of mixing to 75% after 120 minutes of mixing and an opposite effect from that at 20 L/min is thus obtained. In other words, not the whole drug particle mass is affected by mixing time in the same way. The salmeterol fraction that is contained in easily detachable agglomerates increases (Figure 11: coarse, 20 L/min) concurrently with the fraction that is contained in a strongly bound film on the carrier surface (Figure 11: coarse, 60 L/min). Which effect is measured depends on the dispersion efficacy (i.e. flow rate) during the drug detachment experiments. The fact that, at 60 L/min, a plateau in drug detachment is reached suggests that during mixing a dynamic equilibrium is established between drug that is present in the strongly bound film on the carrier surface and drug that is present as more readily detachable drug particles (including agglomerates).

In contrast to the coarse carrier, prolonged mixing with the fine carrier results in a negative effect on drug detachment at 20 L/min. Because the agglomeration of salmeterol is less pronounced with the finer carrier, the positive contribution from this effect is not dominant over any negative contribution from increased compression of the drug onto the carrier surface and redistribution of drug towards active binding sites. The greater dominance of press-on and possibly distribution effects is also apparent at 60 L/min, because no plateau value is reached and the minimum amount of drug detached is lower than with the coarse carrier (42% versus 75%, respectively). Based on the SEM images and apparent solubility data it was concluded that mechanical stress and thus press-on forces on the drug particles are lower for the fine carrier. This effect, which should result in improved drug detachment, is apparently offset by the decrease in agglomeration from the coarse to the fine carrier.

For fluticasone, drug detachment at 20 L/min stays relatively constant on continued mixing with the coarse carrier. This suggests that effects from agglomeration are balanced by press-on effects and distribution effects and are thus less dominant than is the case for salmeterol. This is in agreement with the lower degree of agglomeration that was observed for fluticasone. Also the lower plateau value in drug detachment that is reached at 60 L/min indicates that the dynamic equilibrium between drug being present as detachable agglomerates and as strongly adhering films is shifted towards the latter when changing salmeterol for fluticasone. The effect of mixing time is comparable for both drugs when mixed with the fine carrier. This too is in agreement with the less pronounced difference in their agglomeration behaviour (which for salmeterol is likely restricted by the smaller surface irregularities).

### Practical implications

The results show that the mixing time should be carefully considered for the formulation of carrier-based inhalation powders. The optimal mixing time may vary depending on the formulation purpose and the choice for other, interacting variables. A balance between satisfactory homogeneity and mechanical stability on the one hand and dispersibility on the other may be achieved at relatively short mixing times. However, the effect of mixing time is also most pronounced at the start of mixing, and therefore, a sufficiently robust process may require longer mixing instead. For research purposes it may be desirable to stress press-on effects or agglomeration effects, which can be achieved by prolonged mixing, especially when using a coarse carrier fraction.

The need for a careful consideration of the applied mixing time is further stressed by the fact that the relevance of mixing time is not restricted to the homogeneity and dispersion performance of the powder formulation. The mechanical stress from (prolonged) mixing may influence the solid state of the drug and, with that, its dissolution behaviour (absorption) after inhalation and subsequent deposition in the lungs and the physical and chemical stability of the mixture during storage.

The long mixing times that have been applied in this study may not be representative for the mixing times applied in industrial dry powder inhalation formulation. However, many of the effects that are shown in is this paper may occur at a higher rate when larger batch sizes and higher shear mixing principles are used, as is common for industrial processes. For example, it was shown that 1 hour of mixing with the low shear Turbula blender used in this study (operated at 90 rpm) results in a similar dispersion performance as 5 minutes of mixing with a lab-scale high shear blender (i.e. Picomix operated at 1000 rpm) [27].

### Future perspectives

The overall effect of mixing time on drug detachment is likely to depend on more variables than the ones addressed in this paper. Examples are the dispersion principle, the drug content, the carrier surface roughness, the mixing principle and the mixing intensity. Furthermore, the change in drug detachment is only an indication of the change in the maximum fine particle fraction that can be obtained, depending on the degree of agglomerate dispersion following detachment. Therefore, much is still to be learned from future investigations that address these factors in combination with the right techniques to measure or monitor the relevant powder properties.

## Conclusions

Quantitative and qualitative interactions occur between the effect of mixing time on drug detachment and the type of drug, the carrier size fraction and the flow rate used. This can be satisfactorily explained with a balance of three processes which take place during mixing, i.e., drug (de-) agglomeration, compression onto and (re-)distribution over the carrier surface. A combination of SEM, laser diffraction techniques and the measurement of the apparent solubility of the drug can be used to qualitatively analyse these processes, but an exact quantification requires further improvement of these or the development of other techniques.

## References

[1] Lacey PMC (1997) The mixing of solid particles. Chem Eng Res Des 75, Supplement: S49-S55. doi:10.1016/S0263-8762(97)80004-4.

[2] Hersey JA (1975) Ordered mixing: A new concept in powder mixing practice. Powder Technol 11: 41-44. doi:10.1016/0032-5910(75)80021-0.

[3] Staniforth JN (1981) Total Mixing. Int J Pharm Tech Prod Mfr 2: 7-12.

[4] Staniforth JN (1987) British Pharmaceutical Conference Science Award Lecture 1986: Order out of chaos. J Pharm Pharmacol 39: 329-334. doi:10.1111/j.2042-7158.1987.tb03393.x. PubMed: 2886579. 2886579 10.1111/j.2042-7158.1987.tb03393.x

[5] Kale K, Hapgood K, Stewart P (2009) Drug agglomeration and dissolution - What is the influence of powder mixing? Eur J Pharm Biopharm 72: 156-164. doi:10.1016/j.ejpb.2008.12.015. PubMed: 19347972. 19347972 10.1016/j.ejpb.2008.12.015

[6] de Villiers MM, Lötter AP, van der Watt JG (1993) Influence of surfactants and interactive mixing on the cohesive properties of a poorly wettable solid. Powder Technol 75: 159-165. doi:10.1016/0032-5910(93)80077-N.

[7] de Boer AH, Hagedoorn P, Gjaltema D, Lambregts D, Irngartinger M et al. (2004) The Mode of Drug Particle Detachment from Carrier Crystals in an Air Classifier-Based Inhaler. Pharm Res 21: 2167-2174. doi:10.1007/s11095-004-5171-6. PubMed: 15648247. 15648247 10.1007/s11095-004-5171-6

[8] Travers DN (1975) Some observations on ordered mixing of micronized sodium-bicarbonate with sucrose crystals. Powder Technol 12: 189-190. doi:10.1016/0032-5910(75)80011-8.

[9] de Boer AH, Hagedoorn P, Gjaltema D, Goede J, Kussendrager KD et al. (2003) Air classifier technology (ACT) in dry powder inhalation Part 2. The effect of lactose carrier surface properties on the drug-to-carrier interaction in adhesive mixtures for inhalation. Int J Pharm 260: 201-216. doi:10.1016/S0378-5173(03)00264-3. PubMed: 12842340. 12842340 10.1016/s0378-5173(03)00264-3

[10] Kulvanich P, Stewart PJ (1987) The effect of blending time on particle adhesion in a model interactive system. J Pharm Pharmacol 39: 732-733. doi:10.1111/j.2042-7158.1987.tb06978.x. PubMed: 2890739. 2890739 10.1111/j.2042-7158.1987.tb06978.x

[11] Lam KK, Newton JM (1991) Investigation of applied compression on the adhesion of powders to a substrate surface. Powder Technol 65: 167-175. doi:10.1016/0032-5910(91)80179-M.

[12] Podczeck F (1996) Assessment of the mode of adherence and the deformation characteristics of micronized particles adhering to various surfaces. Int J Pharm 145: 65-76. doi:10.1016/S0378-5173(96)04718-7.

[13] Bridson RH, Robbins PT, Chen Y, Westerman D, Gillham CR et al. (2007) The effects of high shear blending on α-lactose monohydrate. Int J Pharm 339: 84-90. doi:10.1016/j.ijpharm.2007.02.022. PubMed: 17398047. 17398047 10.1016/j.ijpharm.2007.02.022

[14] Shur J, Harris H, Jones MD, Kaerger JS, Price R (2008) The role of fines in the modification of the fluidization and dispersion mechanism within dry powder inhaler formulations. Pharm Res 25: 1631-1640. doi:10.1007/s11095-008-9538-y. PubMed: 18239861. 18239861 10.1007/s11095-008-9538-y

[15] Dickhoff BHJ, de Boer AH, Lambregts D, Frijlink HW (2003) The effect of carrier surface and bulk properties on drug particle detachment from crystalline lactose carrier particles during inhalation, as function of carrier payload and mixing time. Eur J Pharm Biopharm 56: 291-302. doi:10.1016/S0939-6411(03)00109-7. PubMed: 12957644. 12957644 10.1016/s0939-6411(03)00109-7

[16] de Boer AH, Dickhoff BHJ, Hagedoorn P, Gjaltema D, Goede J et al. (2005) A critical evaluation of the relevant parameters for drug redispersion from adhesive mixtures during inhalation. Int J Pharm 294: 173-184. doi:10.1016/j.ijpharm.2005.01.035. PubMed: 15814242. 15814242 10.1016/j.ijpharm.2005.01.035

[17] Le VNP, Robins E, Flament MP (2012) Agglomerate behaviour of fluticasone propionate within dry powder inhaler formulations. Eur J Pharm Biopharm 80: 596-603. doi:10.1016/j.ejpb.2011.12.004. PubMed: 22198291. 22198291 10.1016/j.ejpb.2011.12.004

[18] Dickhoff BHJ, Ellison MJH, de Boer AH, Frijlink HW (2002) The effect of budesonide particle mass on drug particle detachment from carrier crystals in adhesive mixtures during inhalation. Eur J Pharm Biopharm 54: 245-248. doi:10.1016/S0939-6411(02)00082-6. PubMed: 12191698. 12191698 10.1016/s0939-6411(02)00082-6

[19] Begat P, Morton DAV, Staniforth JN, Price R (2004) The Cohesive-Adhesive Balances in Dry Powder Inhaler Formulations II: Influence on Fine Particle Delivery Characteristics. Pharm Res 21: 1826-1833. doi:10.1023/B:PHAM.0000045236.60029.cb. PubMed: 15553229. 15553229 10.1023/b:pham.0000045236.60029.cb

[20] Jones MD, Hooton JC, Dawson ML, Ferrie AR, Price R (2008) An Investigation into the Dispersion Mechanisms of Ternary Dry Powder Inhaler Formulations by the Quantification of Interparticulate Forces. Pharm Res 25: 337-348. doi:10.1007/s11095-007-9467-1. PubMed: 17952568. 17952568 10.1007/s11095-007-9467-1

[21] Elamin AA, Ahlneck C, Alderborn G, Nyström C (1994) Increased metastable solubility of milled griseofulvin, depending on the formation of a disordered surface structure. Int J Pharm 111: 159-170. doi:10.1016/0378-5173(94)00132-4.

[22] Mosharraf M, Nyström C (1999) The effect of dry mixing on the apparent solubility of hydrophobic, sparingly soluble drugs. Eur J Pharm Sci 9: 145-156. doi:10.1016/S0928-0987(99)00043-3. PubMed: 10620727. 10620727 10.1016/s0928-0987(99)00043-3

[23] de Boer AH, Hagedoorn P, Gjaltema D, Goede J, Frijlink HW (2003) Air classifier technology (ACT) in dry powder inhalation: Part 1. Introduction of a novel force distribution concept (FDC) explaining the performance of a basic air classifier on adhesive mixtures. Int J Pharm 260: 187-200. doi:10.1016/S0378-5173(03)00250-3. PubMed: 12842339. 12842339 10.1016/s0378-5173(03)00250-3

[24] Price R (2010) Low and High Energy Blending. Presentation at conference: Lactose as a carrier for inhalation products. Parma.

[25] Hüttenrauch R, Fricke S, Zielke P (1985) Mechanical Activation of Pharmaceutical Systems. Pharm Res 2: 302-306. doi:10.1023/A:1016397719020. 24271128 10.1023/A:1016397719020

[26] Mosharraf M, Nyström C (2003) Apparent Solubility of Drugs in Partially Crystalline Systems. Drug Dev Ind Pharm 29: 603-622. doi:10.1081/DDC-120021310. PubMed: 12889779. 12889779 10.1081/ddc-120021310

[27] Cordts E, Grasmeijer F, Van der Wel P, Dekens B, De Boer AH et al. (2012) The influence of mixing time and intensity on blend homogeneity and dispersion performance. Drugs Deliv Lungs 23 Edinburgh. pp. 146-149

