# Correction: Mixing Time Effects on the Dispersion Performance of Adhesive Mixtures for Inhalation

**DOI:** 10.1371/annotation/7302762a-a6fe-4fcd-8656-36d8b7c259a5

**Published:** 2013-12-31

**Authors:** Floris Grasmeijer, Paul Hagedoorn, Henderik W. Frijlink, H. Anne de Boer

In Table 2, the data in row "600 min" shifted four columns to the left and the data in row "780 min" shifted six columns to the left. Please see the corrected Table 2 here: http://plosone.org/corrections/pone.0069263.t002.cn.tif

